# Multi-Object Detection Method in Construction Machinery Swarm Operations Based on the Improved YOLOv4 Model

**DOI:** 10.3390/s22197294

**Published:** 2022-09-26

**Authors:** Liang Hou, Chunhua Chen, Shaojie Wang, Yongjun Wu, Xiu Chen

**Affiliations:** 1 Department of Mechanical and Electrical Engineering, Xiamen University, Xiamen 361102, China; 2 Shenzhen Research Institute of Xiamen University, Shenzhen 518057, China

**Keywords:** construction machinery, swarm operation scenario, multi-object detection, YOLOv4 model

## Abstract

To handle the problem of low detection accuracy and missed detection caused by dense detection objects, overlapping, and occlusions in the scenario of complex construction machinery swarm operations, this paper proposes a multi-object detection method based on the improved YOLOv4 model. Firstly, the K-means algorithm is used to initialize the anchor boxes to improve the learning efficiency of the depth features of construction machinery objects. Then, the pooling operation is replaced with dilated convolution to solve the problem that the pooling layer reduces the resolution of feature maps and causes a high missed detection rate. Finally, focus loss is introduced to optimize the loss function of YOLOv4 to improve the imbalance of positive and negative samples during the model training process. To verify the effectiveness of the above optimizations, the proposed method is verified on the Pytorch platform with a self-build dataset. The experimental results show that the mean average precision(mAP) of the improved YOLOv4 model for multi-object detection of construction machinery can reach 97.03%, which is 2.16% higher than that of the original YOLOv4 detection network. Meanwhile, the detection speed is 31.11 fps, and it is reduced by only 0.59 fps, still meeting the real-time requirements. The research lays a foundation for environment perception of construction machinery swarm operations and promotes the unmanned and intelligent development of construction machinery swarm operations.

## 1. Introduction

As a research hotspot in the field of machine vision, object detection has been of wide concern to researchers for a long time [1]. The purpose of object detection is to find several objects in the complex background of the image, give an accurate object frame, and determine the category of the object [2]. Traditional object detection usually includes three stages: (1) using windows of different sizes to slide on the image to determine candidate regions; (2) extracting features from the candidate regions; and (3) using a trained classifier for classification. Traditional detection methods such as the scale-invariant feature transform (SIFT) algorithm [3], speeded-up robust features (SURF) algorithm [4], Viola–Jones algorithm [5,6], etc., need to extract features and develop classifiers according to the characteristics of the detection target, leading to poor robustness, long time consumption, and low reusability. Meanwhile, the algorithm uses the exhaustive sliding window strategy, which generates many redundant windows and affects the detection speed. With the rapid development of deep learning in recent years, breakthroughs have been made in object detection. The detection methods based on deep learning can well achieve feature learning and classification with high detection accuracy and fast detection speed. At present, the object detection models based on deep learning are mainly divided into two categories: one is two-stage object detection methods based on region proposals, such as region-convolutional neural network (RCNN) [7], Fast-RCNN [8], Faster-RCNN [9], etc. This type of model consists of two steps: candidate region generation, object classification and coordinate regression by the neural network. It features high accuracy and slow speed; the other is one-stage object detection methods based on regression, such as single shot multi-box detector (SSD) [10], you only look once (YOLO) series [11,12,13,14,15], etc. The detection process of this type of model has only one stage, i.e., generating candidate regions and object categories at the same time, which greatly shortens the time for object detection.

In the field of construction machinery, the object detection models based on deep learning are widely used in the monitoring, tracking, and danger warning of the vehicles and personnel at the job site, which helps to improve construction efficiency, reduce labor costs, and ensure construction safety. Bo Xiao et al. [16] adopted the deep learning illumination enhancement method to improve the lighting conditions in nighttime videos, and exploited a convolutional neural network (CNN) feature extractor as the appearance model to distinguish construction machines, which improved the productivity and safety of night construction. Zhang Shen et al. [17] adopted an improved Faster-RCNN model to detect construction machinery, solve the problem of transmission line tripping caused by construction machinery intrusion, and reduce manual maintenance costs and economic losses. Bo Xiao et al. [18] used the YOLOv3 model to build a construction machinery tracker, which automatically tracks construction machinery in the video to monitor construction safety and project progress in real-time. Weili Fang et al. [19]. proposed an improved Faster-RCNN model to detect workers and heavy equipment in construction sites in real-time to improve safety and productivity. To sum up, object detection is indispensable to the field of construction machinery and is an important basis for the intelligence of construction machinery, which can ensure efficiency and safety in harsh or dangerous environments. Therefore, the research on the object detection model of construction machinery will help to promote the unmanned and intelligent development of construction machinery swarm operations.

At present, the YOLO series is a hotspot in object detection. From YOLOv1 proposed by Redmon in 2016 to YOLOX in 2021, the performance has been continuously increased. The YOLOv4 model combines previous research techniques with appropriate innovation to achieve excellent speed and accuracy [20]. It is widely used in practice and has become a mature object detection technique. In order to solve the problems of low face recognition accuracy and poor real-time performance when wearing a mask, Yu et al. [21] improved the cross-stage-partial darknet53 (CSPDarkNet53) and path aggregation network (PANet) structures of the YOLOv4 model, but they failed to consider the situation of wearing a mask when the light was insufficient; Jiang et al. [22]. optimized the backbone of the YOLOv4-tiny model to reduce network parameters, which greatly improves the detection speed of the model. Meanwhile, they proposed a real-time object detection model suitable for embedded devices. The disadvantage is that the detection accuracy is slightly reduced; Hu [23] modified the feature map with dense connections and de-redundancy operations to improve YOLOv4 and applied the model to detect and monitor the consumption of feed pellets in aquaculture in real-time, which effectively improves the detection accuracy and speed of the model. Fu et al. [24] improved the detection effect of self-built marine object datasets by adding a convolutional attention module to the YOLOv4 model, but the detection speed of the model is slow. Guo et al. [25] replaced the activation functions of the YOLOv4 backbone with other activation functions, to solve the problems of large labor cost and high risk in the detection of railway track components, which improved the detection accuracy and promoted railway safety. To enable the orchard robot to detect apples quickly and accurately, Wu et al. [26] replaced the backbone of YOLOv4 network with EfficientNet. The improvement of the YOLOv4 model in the above literature focuses on the backbone network, which has an important impact on improving the detection effect of the model. However, the influence of the pooling layer and loss function in the model on the detection effect is ignored.

For construction machinery swarm operations, this paper proposes a multi-object detection model based on improved YOLOv4, which combines the K-means clustering algorithm, dilated convolution, and focus loss function. In order to train and test the model, this paper constructed a self-built dataset. Compared with the original model, the improved model has higher detection accuracy and robustness for overlapping and occluded targets, and achieves a similar processing speed. This research is expected to provide inspiration for other applications in environmental perception of construction machinery swarm operations.

The rest of this paper is organized as follows: Section 2 briefly introduces the YOLOv4 detection model and describes the improvements of the model; Section 3 presents the training and parameter adjustment of the improved model; Section 4 discusses the actual detection results of the improved model. Finally, the research work of this paper is concluded.

## 2. Improved YOLOv4 Network Model

### 2.1. The YOLOv4 Network Model

**Figure 1:**
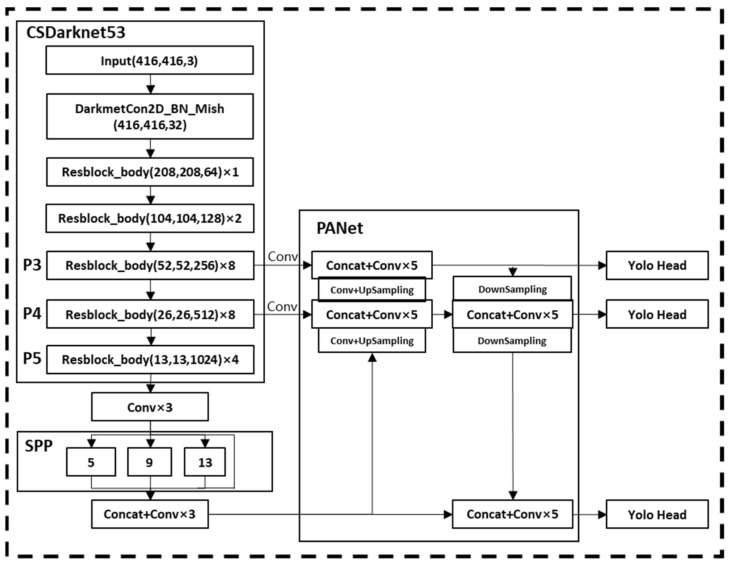
YOLOv4 network structure.

The YOLOv4 model consists of three parts: the backbone network that extracts features, the neck structure that transmits features to the detection network, and the head structure responsible for detection. The backbone network of YOLOv4 is CSPDarknet53 [27], which is improved based on Darknet53 by borrowing the idea of cross-stage partial network (CSPNet). This solves the problem of repeating gradient information in network optimization in the backbone framework of large-scale convolutional neural networks and reduces the size of the model. Attributed to this, the detection speed and accuracy are improved. The neck structure is composed of the spatial pyramid pooling network (SPP-Net) [28] and the path aggregation network (PANet) [29]. The SPP structure is used to increase the receptive field of the network and obtain richer feature information. The PAN structure performs parameter aggregation to adapt to different levels of object detection. For the detection head part, YOLOv4 still uses the detection head of the YOLOv3 model. The structure of YOLOv4 is shown in Figure 1:

In addition to the above improvements, YOLOv4 also adopts a large number of optimizations, including weighted residual connections (WRC) [30], cross-mini-batch normalization (CmBN) [31], Mish activation function [32], Mosaic data enhancement, DropBlock regularization [33], CIoU Loss [34], etc.

### 2.2. Improved Network Model

**Figure 2:**
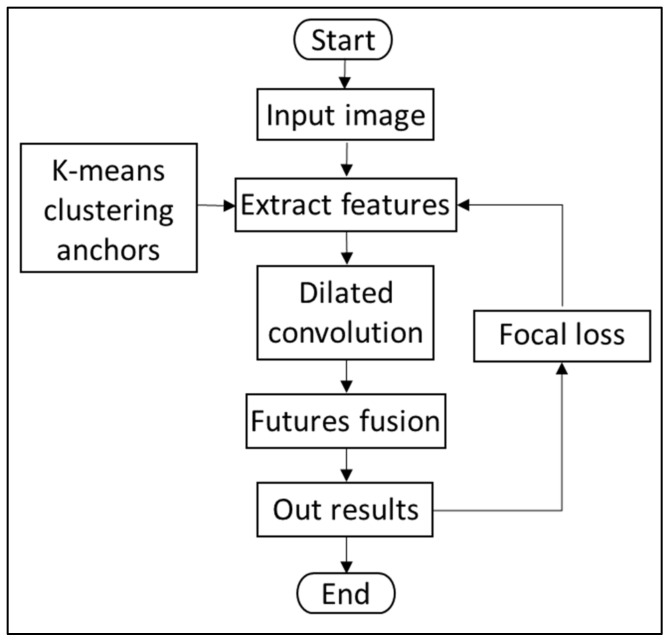
Flow chart of the improved network.

In this paper, objects such as people, loaders, excavators, and trucks in the scenario of construction machinery swarm operations are taken as detection objects. Since dense detection objects, overlapping, and occlusion of these detection objects lead to low detection accuracy and missed detection, this paper makes the following improvements to YOLOv4: (1) use the K-means algorithm to initialize the anchor boxes of objects to improve the learning efficiency of the deep features of the construction machinery objects; (2) replace the pooling layer in the neck SPP structure with dilated convolution to handle the problem of image resolution degradation due to max-pooling; and (3) introduce the focal loss function to solve the problem of low detection accuracy caused by the imbalance of positive and negative samples in the detection model. The specific flow of the improved model is shown in Figure 2. After the image is input, the clustered anchor boxes are used to improve the efficiency of image feature extraction; then, the features are further extracted through dilated convolution and processed by feature fusion; finally, the prediction result is output. The loss function adjusts the learning parameters of the network through the output results.

#### 2.2.1. K-Means Algorithm

Anchor boxes (anchors) have an important impact on the accuracy of the object detection model. Suitable anchors provide prior knowledge for the model before training, which can improve the learning efficiency and convergence speed of the model. In object detection models, anchors can be designed by manual work and machine learning methods. The anchors of object detection models such as SSD and Faster-RCNN are designed artificially. Such anchors may not be suitable for training datasets. If the size of the anchors is quite different from the object, then it will reduce the detection effect of the model and even produce gradient explosion. The anchors of the object detection models such as YOLOv3 and YOLOv4 are designed by machine learning methods. The anchors of YOLOv4 are clustered by the K-means [35] algorithm on the PASCALL_VOC and COCO datasets. Though these two datasets contain a wide variety of object sizes, they are not the most suitable dataset for the scenario considered in this paper. Therefore, this paper uses the K-means algorithm to cluster the bounding boxes (Boxes) to generate suitable anchors.

The standard K-means algorithm uses Euclidean distance as a metric. However, the model in this paper only cares about the IOU (Intersection Over Union) of anchors and Boxes when clustering boxes, so taking IOU as the metric of K-means is more appropriate. The model in this paper needs nine anchors for the prediction of each grid, so the number of clusters k in the K-means algorithm is 9. Finally, the K-means algorithm is used to perform cluster analysis on the construction machinery dataset in this paper, and the sizes of the nine anchors obtained are 16 × 37, 42 × 68, 58 × 145, 97 × 92, 101 × 203, 165 × 149, 168 × 297, 253 × 220, and 342 × 333.

#### 2.2.2. Dilated Convolution

The SPP structure in YOLOv4 uses a max-pooling layer of sizes 5, 9, and 13 to increase the receptive field and improve the detection performance of the model. However, using the pooling operation will reduce the resolution of the feature map, lose spatial information, reduce the detection ability of occluded objects, and increase the missed detection rate of the model.

**Figure 3:**
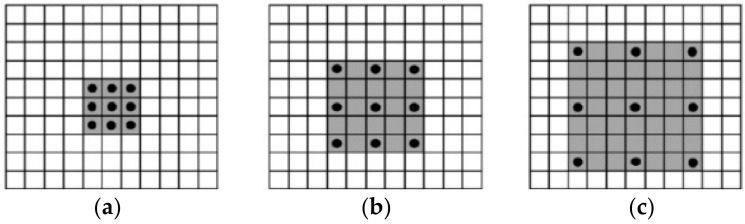
3 × 3 dilated convolution with different dilated rates. (**a**) dilated rates = 1, (**b**) dilated rates = 2, (**c**) dilated rates = 3.

To increase the receptive field while ensuring the resolution of the feature map, and improve the detection ability of the network for occluded objects, dilated donvolution (DC) [36] is introduced into the model proposed in this paper. Unlike ordinary convolutions, dilated convolutions contain a “Dilation Rate” hyperparameter, which defines the number of padding 0 s in the convolution kernel. Figure 3 shows the dilated convolution with dilated rates of 1, 2, and 3 respectively, and the dilated convolution with a dilated rate of 1 is the ordinary convolution. The receptive field of the dilated convolution with different convolution kernels is calculated as follows:

(1) $$\;k{}^{\prime }=k+\left(k-1\right)\times \left(d-1\right)$$

where $\;k{}^{\prime }$ is the equivalent convolution kernel size, k is the convolution kernel size of the dilated convolution, and d is the dilated rate.

(2) $${\mathrm{RF}}_{i+1}={\;\mathrm{RF}}_{i}+\left(k-1\right)\times S_{i}$$

where ${\mathrm{RF}}_{i+1}$ represents the receptive field of the current layer, ${\mathrm{RF}}_{i}$ represents the receptive field of the previous layer, and $S_{i}$ is the step product of all previous layers.

Therefore, it can be seen from Figure 3 that the receptive field of 3 × 3 dilated convolution with d = 2 and 3 is 5 and 7. Compared with ordinary convolution, it has a larger receptive field with the same number of parameters and computation.

Since the calculation method of dilated convolution is similar to the checkerboard format, the convolution results of a certain layer come from an independent set of the previous layer, and there is no correlation between the convolution results in the current layer, which results in the loss of local information. To handle this problem, this paper adopts hybrid dilated convolution (HDC) by combining dilated convolutions with different dilated rates.

**Figure 4:**
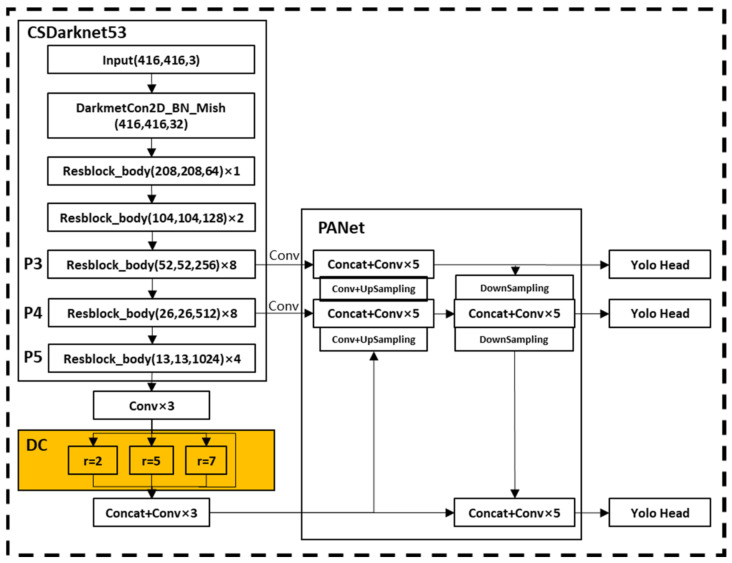
Improved network structure through the dilated convolution.

In this paper, referring to the SPP structure of YOLOv4, the max-pooling layers with sizes of 5, 9, and 13 are replaced with dilated convolutions with dilated rates of 2, 5, and 7 to obtain feature information at different scales. The activation function of dilated convolution adopts the Mish function, which can make the model obtain better accuracy and generalization. Based on these optimizations, the improved network structure is shown in Figure 4.

#### 2.2.3. Focal Loss

The imbalance of positive and negative samples in the training process is one of the causes of low detection accuracy of the network model. In a picture of construction machinery operation, most of the objects to be detected only occupy a small part, and the rest are backgrounds. Therefore, most of the anchors generated by the model are negative samples (i.e., background), which makes negative samples have a much larger contribution than positive samples. In this case, the model tends to consider that most positions do not contain objects, resulting in low model training efficiency and reduced accuracy. To address this issue, this paper introduces the focal loss [37] function to improve the loss calculation of the model and improve the problem of unbalanced positive and negative samples.

The confidence and category loss functions of YOLOv4 are both binary cross-entropy functions. The calculation of binary cross-entropy loss (L) is shown in formula (3):

(3) $$L=-\mathrm{ylogy}{}^{\prime }-\left(1-y\right)\log\left(1-y{}^{\prime }\right)\;=\left\{\begin{array}{c} -\mathrm{logy}{}^{\prime },\;\;\;\;\;\;y=1\\ -\log\left(1-y{}^{\prime }\right),\;\;y=0 \end{array}\right.$$

where $\;y{}^{\prime }$ is the predicted value of the model, y is the ground truth value, and 1, 0 represent positive and negative samples, respectively. It can be seen from Formula (3) that the contribution factor of positive and negative samples to the loss is the same. The focal loss makes the following improvements to the binary cross-entropy function ($L_{\mathrm{FL}}$):

(4) $${\;L}_{\mathrm{FL}}=\left\{\begin{array}{c} -\alpha {\left(1-\;y{}^{\prime }\right)}^{\gamma }\mathrm{logy}{}^{\prime },\;\;\;\;\;\;y=1\\ -\left(1-\alpha \right){y{}^{\prime }\;}^{\gamma }\log\left(1-\;y{}^{\prime }\right),\;y=0 \end{array}\right.$$

It can be seen from Formula (4) that the focal loss function adds coefficients α and (1 − α) before the positive and negative samples, respectively. By adjusting the value of α, the contribution of the positive samples to the loss can be changed, and the imbalance between positive and negative samples can be alleviated. The default value of α is 0.25. Meanwhile, the modulation coefficient ${\left(1-\;y{}^{\prime }\right)}^{\gamma }$ and ${\;y{}^{\prime }}^{\gamma }$ are added before the positive and negative samples respectively, so that the model pays more attention to the difficult-to-classify samples. Take the positive sample as an example: when the value of ground truth value (y) is 1, the closer the predicted value $\;y{}^{\prime }$ is to 1, the easier the sample is to predict, and ${\left(1-\;y{}^{\prime }\right)}^{\gamma }$ is smaller; when the closer the predicted value $\;y{}^{\prime }$ is to 0, it means the sample is difficult to predict, and ${\left(1-\;y{}^{\prime }\right)}^{\gamma }$ is bigger. This is the same for negative samples. γ is a constant greater than 0 and defaults to 2. The confidence loss (${\;L}_{\mathrm{conf}\;}$) and category loss (${L\;}_{\mathrm{class}\;}$) functions of the model are replaced with the focal loss, as shown in Equations (5) and (6):

(5) $${\;L\;}_{\mathrm{conf}\;}=\;\;\;\;\;\;\;\;\;\;\;\;\;\;\;\;\;\;\;\;\;\;\;-\sum_{i=0}^{S^{2}}\sum_{j=0}^{B}I_{ij}^{\mathrm{obj}}\left[{\hat{C}}_{i}^{j}\alpha {\left(1-C_{i}^{j}\right)}^{\gamma }\log\left(C_{i}^{j}\right)+\left(1-{\hat{C}}_{i}^{j}\right)\log\left(1-C_{i}^{j}\right)\right]\;-\sum_{i=0}^{S^{2}}\sum_{j=0}^{B}I_{ij}^{\mathrm{noobj}}\left[{\hat{C}}_{i}^{j}\log\left(C_{i}^{j}\right)+\left(1-{\hat{C}}_{i}^{j}\right)\left(1-\alpha \right){\left(C_{i}^{j}\right)}^{\gamma }\log\left(1-C_{i}^{j}\right)\right]$$

(6) $${L\;}_{\mathrm{class}\;}\;=\;\sum_{i=0}^{S^{2}}\;I_{ij}^{\mathrm{obj}}\;\;\underset{c\in classes}{\sum }\left[\alpha {\left(1-{\hat{p}}_{i}^{j}\left(c\right)\right)}^{\gamma }\log\left({\hat{p}}_{i}^{j}\left(c\right)\right)+\left(1-\alpha \right){\hat{p}}_{i}^{j}{\left(c\right)}^{\gamma }\log\left(1-{\hat{p}}_{i}^{j}\left(c\right)\right)\right]$$

where $S^{2}$ is the number of grids divided from the picture; *B* represents the number of anchors generated by each grid; $I_{ij}^{\mathrm{obj}\;}$ and $I_{ij}^{\mathrm{noobj}\;}$ jointly determine the positive and negative samples. When $I_{ij}^{\mathrm{obj}\;}$ is 1 and $I_{ij}^{\mathrm{noobj}\;}$ is 0, it represents a positive sample; otherwise, it is a negative sample; ${\hat{C}}_{i}^{j}$ represents the truth confidence and takes a value of 1 or 0; $C_{i}^{j}$ is the prediction confidence value of the sample; ${\hat{p}}_{i}^{j}$ represents the true class, and $p_{i}^{j}$ represents the probability of the predicted class. This paper does not optimize the position loss of the model. Based on the improvements of K-means and dilated convolution, the performance under different parameters of focal loss is shown in Table 1, and the evaluation index is the mean average precision (mAP). During the experiment, the parameter γ was set to 2 and 1.5, respectively, and the performance of the model under different α values was compared. It can be seen from Table 1 that when the value of α was 0.3, the highest mAP value was obtained. Then, the value of α was set to 0.3, and the performance under different values of γ was compared. Finally, the focus loss parameter value was selected as (1.5, 0.3).

## 3. Model Training and Tuning

### 3.1. Experimental Dataset

#### 3.1.1. Dataset Acquisition

**Figure 5:**
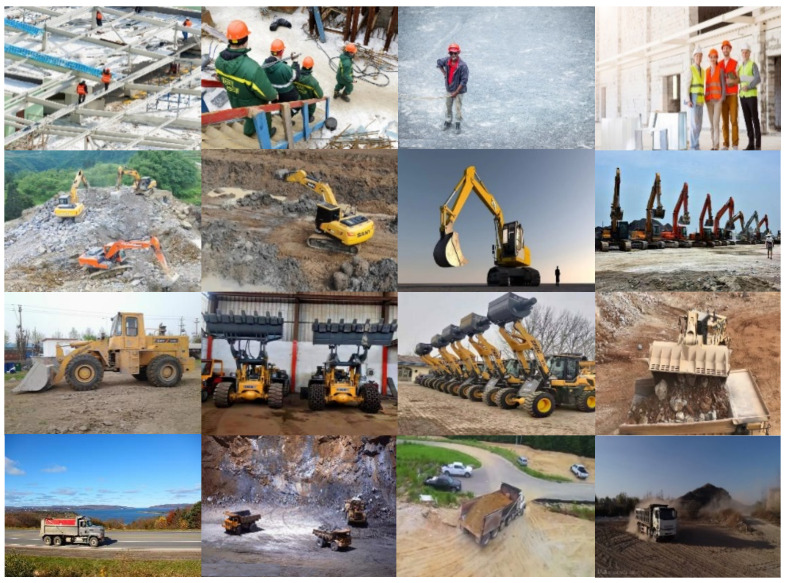
Partial dataset example.

**Figure 6:**
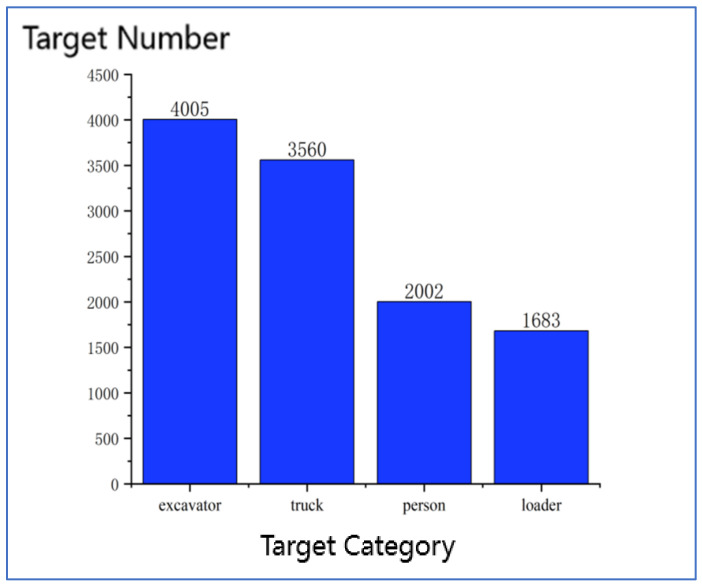
Dataset size of different detection objects.

At present, there is no public dataset of construction machinery swarm operations. To train and test the model, this paper constructed a self-built dataset that includes people, loaders, excavators, and trucks in the swarm operation scene of construction machinery. All images were collected in two ways: online collection and onsite collection. For online collection, construction images and videos were downloaded from photo-sharing and video-sharing websites using python crawlers. In general, construction videos change slowly, videos collected from websites are downloaded and converted to images at a rate of one image every 5 s. And make sure each image included at least one target machine. For onsite collection, images and videos of real construction sites were collected in two ways: using flying unmanned autonomous vehicles and manually taking photos. All videos collected online and onsite have been converted to images in JPEG format. Some example detection objects and their quantities are shown in Figure 5 and Figure 6. The pictures in the dataset contain different angles, environments, distances, poses, and numbers of objects, which are consistent with the object state in the actual situation. Meanwhile, to make the trained network better meet the detection requirements in complex construction machinery operation scenarios and enhance the robustness of the model, online data enhancement is adopted in the training process, and the images are randomly scaled, flipped, and gamut distorted, etc.

#### 3.1.2. Calibration of the Dataset

In this paper, the dataset was calibrated using the LabelImg tool, and the labeling format is the VOC data format. After calibration, a xml file containing the category and location information of the detection object was generated. Before training, the labeled dataset was randomly divided into training-verification set and test set at the ratio of 9:1. Then, the pictures in the training-verification set were randomly divided into the training set and verification set at the ratio of 9:1.

### 3.2. Experimental Platform and Parameters

The configuration of the experimental platform is as follows: Intel(R) Xeon(R) Gold 6230R CPU, 128 GB memory, NVIDIA RTX A5000 graphics card, and Win10 operating system. Cuda11.0 and Cudnn8.0.5.39 were used to accelerate the training process, and data processing was realized with Python3.7 and Pytorch1.7.1.

**Figure 7:**
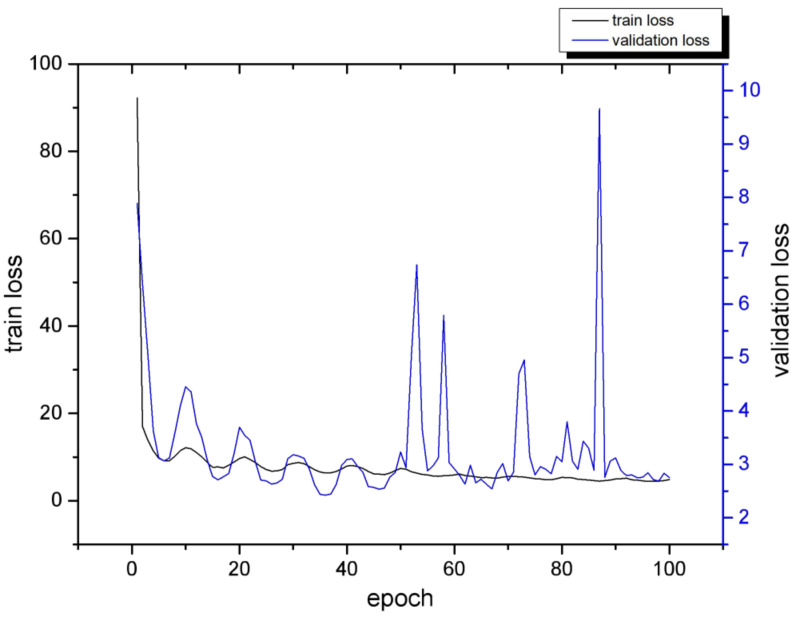
Model loss curves for different Epochs.

The network parameters were configured as follows. Considering that if the network is trained from scratch, the setting of the weights is too random and time-consuming, and the feature extraction effect is not good. Thus, this paper adopted the weights of the VOC2007 dataset for pre-training. Also, the loss curve was used to determine the appropriate number of training rounds (Epoch) to obtain a network model with strong generalization ability. For an ideal loss curve, both the training loss and validation loss converge, and the two values are not much different. During the training process, the number of Epochs was set to 100, and the obtained loss curve is shown in Figure 7. It can be seen that when the number of Epochs is 50, the two loss values converge and have similar sizes; after 50 Epochs, the validation loss increases because the model is overfitting and the generalization ability is reduced. Therefore, the number of training rounds of the model in this paper was set to 50 Epoch. The training consists of two stages: the freezing stage of 25 Epochs with a learning rate of 1 × 10^−3^ and the unfreezing stage of 25 Epochs with a learning rate of 1 × 10^−4^. The training techniques of Mosaic data augmentation, cosine annealing learning rate, and label-smoothing with a value of 0.005 were also applied. According to the network parameters and GPU memory size, the batch size of the freezing stage was set to 32, and that of the unfreezing stage was set to 16.

## 4. Experimental Results and Analysis

### 4.1. Evaluation Metrics for Model Performance

To evaluate the training effect of the model, the commonly used evaluation metrics for object detection, i.e., the mean average precision (mAP), the frame per second (FPS), and the overall model performance value (F1), were used as the evaluation indicators of the model. The formula for calculating F1 is:

(7) $$\left\{\begin{array}{c} P=\frac{T_{P}}{T_{P}+F_{P}}\times 100\%\\ R=\frac{T_{P}}{T_{P}+F_{N}}\times 100\%\\ F1=\frac{2\mathrm{PR}}{P+R} \end{array}\right.$$

where P is the precision rate; R is the recall rate; $T_{P}$/$F_{P}$ is the number of positive (negative) samples that are classified as positive; $F_{N}$ is the number of positive samples that are classified as negative; F1 is the harmonic mean of the precision rate P and the recall rate R. The closer F1 is to 1, the better the model optimization is.

### 4.2. Comparative Analysis of Experimental Results

To further analyze the influence of different improvement strategies on the object detection effect in the scenario of complex construction machinery operations, a comparative experiment was conducted under the condition that score-threshold was set to 0.5, and the experimental results are shown in Table 2. In Table 2, “√” indicates that the corresponding strategy is adopted in this experiment, and “×” indicates that the strategy is not adopted. It can be seen from Table 2 that the F1 value of the improved network is 0.94, with an increase of 0.002; the mAP value is 97.03%, with an increase of 2.16%; the FPS value is 31.11 frames/s, with a decrease of 0.59 frames/s, which can meet the speed requirements of engineering applications.

Meanwhile, to evaluate the effectiveness of this model, the same dataset was applied to the Faster-RCNN with Restnet50 and visual geometry group (Vgg), the YOLOv4, and the SSD with mobilenetv2 and Vgg. The comprehensive performance of the six network models was analyzed, and the comparison results are shown in Table 3. It can be seen from Table 3 that the improved model achieves the highest mAP value, and its FPS value reaches 31.11 frames/s, which can meet the requirements of engineering applications.

**Figure 8:**
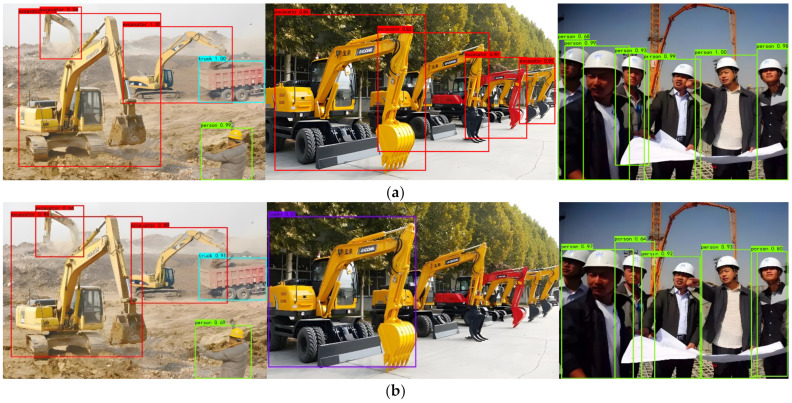
Detection results of densely overlapping objects. (**a**) The improved YOLOv4 model. (**b**) The Original YOLOv4 model.

To test the actual detection effect of the network on the detection object, the two networks with the largest mAP value were selected for comparison. The detection of densely occluded objects is always a major difficulty in the field of object detection, so three images with densely overlapping objects were selected. The detection results are shown in Figure 8:

It can be seen from Figure 8 that the detection ability of the improved model is much better than that of the original Yolov4 model. Except that a distant excavator with large occlusion in the second picture is not correctly detected, the other objects are correctly detected, and the object confidence values are greater than those of the original Yolov4 model.

**Figure 9:**
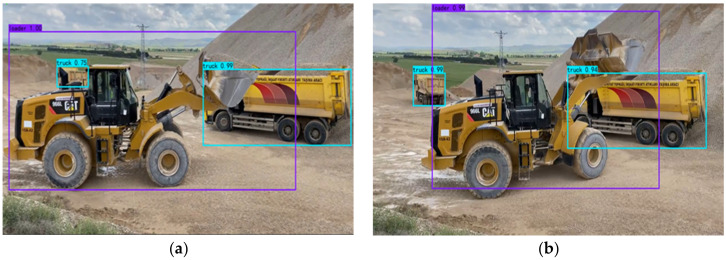

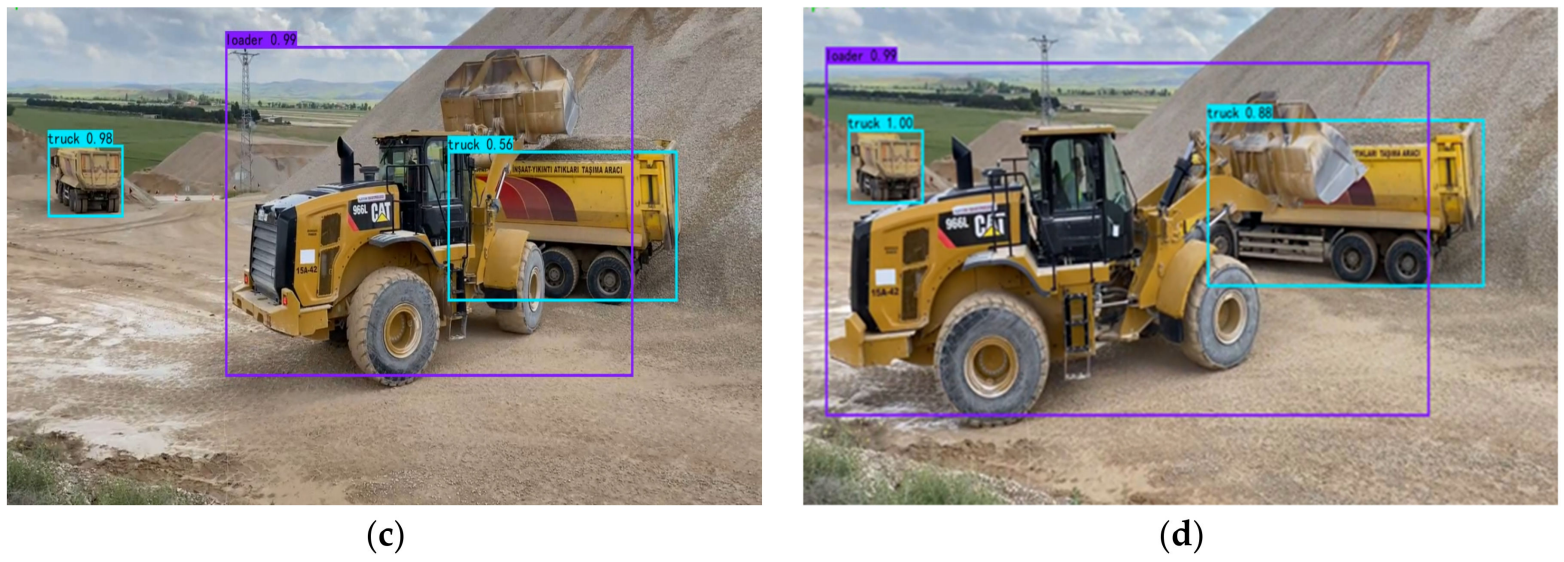
Example improved model detection results for video 1 under occlusions. (**a**) Frame 2229, (**b**) Frame 2278, (**c**) Frame 2331, (**d**) Frame 2930.

Then the detection ability of the improved model was also tested on construction videos that contained occlusions (Video 1 was released by Caterpillar. Video 2 was released by XCMG Construction Machinery). Figure 9 shows an example of the improved model’s detection results under an occlusion scenario in video 1, in which a wheel loader is working with a dump truck for sand-loading activity. In (a) and (b), the wheel loader is moving towards the truck with some occlusion. The wheel loader then moves away from the truck in (c) and (d), and half the pixel area of the truck was occluded by the wheel loader in (c). During this period, the improved model produces precise detection results in this difficult scenario (Figure 9).

**Figure 10:**
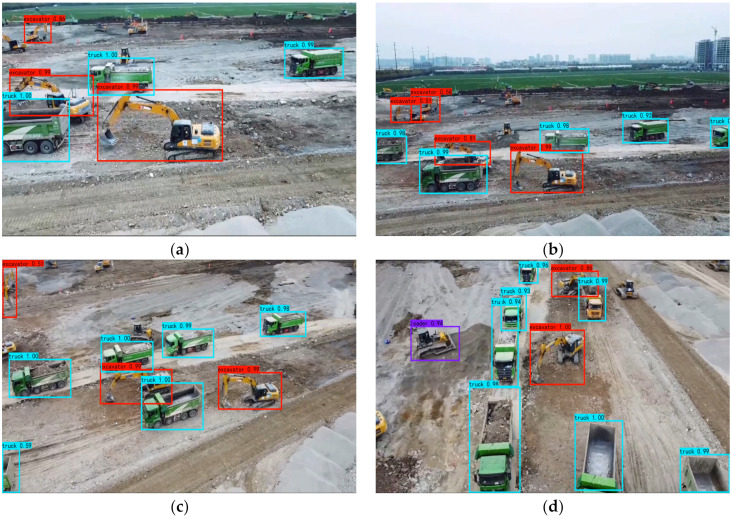
Example improved model detection results for video 2 under occlusions. (**a**) Frame 527, (**b**) Frame 581, (**c**) Frame 779, (**d**) Frame 928.

Figure 10 shows an example of the improved model’s detection results in video 2, in which many construction machines are working together with some occlusion. From (a) to (d) in Figure 10, the improved model can produce stable detection results, although the appearance of these construction machines changed rapidly.

## 5. Conclusions

To handle the problem of low accuracy caused by overlapping and occlusion of multi-object in the scenario of complex construction machinery operations, this paper proposes an object detection model based on the improved Yolov4 model. The advantages of the model are as follows: the anchors are initialized by the K-means algorithm to accelerate the convergence of the model and improve the average accuracy; the pooling pyramid layer of YOLOv4 is replaced with the dilated convolution layer to ensure the resolution of the feature map, reduce the missed detection rate, and increase the receptive field of the network, obtain more global feature information, and have a strong detection ability for overlapping and occluded targets; the focus loss is introduced address the imbalance of positive and negative samples in the dataset; finally, online data enhancement methods such as image scaling, flipping, and color gamut distortion are used during training to enhance the robustness of the model, so that it can adapt to the complex detection environment of construction machinery.

The final experimental results show that the improved detection model achieves a mAP of 97.03%, which is 2.16% higher than that before the improvement. The actual detection performance is good; the improved model can produce precise detection results in occluded scenarios. In addition, the FPS is 31.11 frames/s, which is only reduced by 0.59 frames/s. This is because the improved model introduces more parameters, and parameter tuning takes a certain amount of time, but the detection model can meet the speed requirements of engineering applications. This research is expected to lay the foundation for other applications in environmental perception of construction machinery swarm operations, and promote unmanned and intelligent development of construction machinery swarm operations.

## Figures and Tables

**Table 1:** Comparison of the mAP of the model under different focal loss parameters.

Parameter α	Parameter γ	mAP (%)
0.25	2	96.39
0.3	2	96.62
0.35	2	96.47
0.4	2	96.45
0.45	2	96.27
0.5	2	96.32
0.25	1.5	96.75
0.3	1.5	97.03
0.35	1.5	96.46
0.4	1.5	96.32
0.45	1.5	96.13
0.5	1.5	96.24
0.3	1	96.47
0.3	0	95.75

**Table 2:** Comparison of the mAP of the model under different improvement strategies.

Model	K-Means	Focal Loss	Dilated Convolution	F1	mAP (%)	FPS
YOLOv4	×	×	×	0.938	94.87	31.70
	√	×	×	0.938	96.15	32.11
	×	√	×	0.93	95.96	30.17
	×	×	√	0.935	95.81	30.14
	√	×	√	0.94	95.68	30.86
	×	√	√	0.928	95.37	30.34
	√	√	×	0.938	96.14	30.49
	√	√	√	0.94	**97.03**	31.11

**Table 3:** Performance comparison of different models.

Model	AP (%)				F1	mAP (%)	FPS
	Loader	Excavator	Truck	Person			
Faster-RCNN (Resnet50)	97.21	95.20	92.11	86.43	0.75	93.00	20.94
Faster-RCNN (Vgg)	96.77	94.14	90.80	84.54	0.68	91.56	21.32
SSD (mobilenetv2)	96.08	91.60	86.73	71.05	0.82	86.37	71.37
SSD (Vgg)	99.25	96.03	92.82	83.38	0.897	92.87	72.04
YOLOv4	98.13	97.41	91.25	92.69	0.938	94.87	31.70
Improved YOLOv4	99.79	97.39	96.61	94.33	0.94	97.03	31.11
